# Cartilage-targeting poly(ethylene glycol) (PEG)-formononetin (FMN) nanodrug for the treatment of osteoarthritis

**DOI:** 10.1186/s12951-021-00945-x

**Published:** 2021-07-03

**Authors:** Wei Xiong, Qiumei Lan, Xiaonan Liang, Jinmin Zhao, Hanji Huang, Yanting Zhan, Zainen Qin, Xianfang Jiang, Li Zheng

**Affiliations:** 1 Guangxi Engineering Center in Biomedical Materials for Tissue and Organ Regeneration, The First Affiliated Hospital of Guangxi Medical University, Nanning, 530021 China; 2 Department of Orthopedics Trauma and Hand Surgery, The First Affiliated Hospital of Guangxi Medical University, Nanning, 530021 China; 3 Guangxi Collaborative Innovation Center for Biomedicine, The First Affiliated Hospital of Guangxi Medical University, Nanning, 530021 China; 4 Department of Oral Radiology, Guangxi Medical University College of Stomatology, Nanning, 530021 China

**Keywords:** Formononetin, PEG, Cartilage-targeting, Osteoarthritis

## Abstract

Intra-articular (IA) injection is an efficient treatment for osteoarthritis, which will minimize systemic side effects. However, the joint experiences rapid clearance of therapeutics after intra-articular injection. Delivering system modified through active targeting strategies to facilitate localization within specific joint tissues such as cartilage is hopeful to increase the therapeutic effects. In this study, we designed a nanoscaled amphiphilic and cartilage-targeting polymer-drug delivery system by using formononetin (FMN)-poly(ethylene glycol) (PEG) (denoted as PCFMN), which was prepared by PEGylation of FMN followed by coupling with cartilage-targeting peptide (CollBP). Our results showed that PCFMN was approximately regular spherical with an average diameter about 218 nm. The in vitro test using IL-1β stimulated chondrocytes indicated that PCFMN was biocompatible and upregulated anabolic genes while simultaneously downregulated catabolic genes of the articular cartilage. The therapeutic effects in vivo indicated that PCFMN could effectively attenuate the progression of OA as evidenced by immunohistochemical staining and histological analysis. In addition, PCFMN showed higher intention time in joints and better anti-inflammatory effects than FMN, indicating the efficacy of cartilage targeting nanodrug on OA. This study may provide a reference for clinical OA therapy.
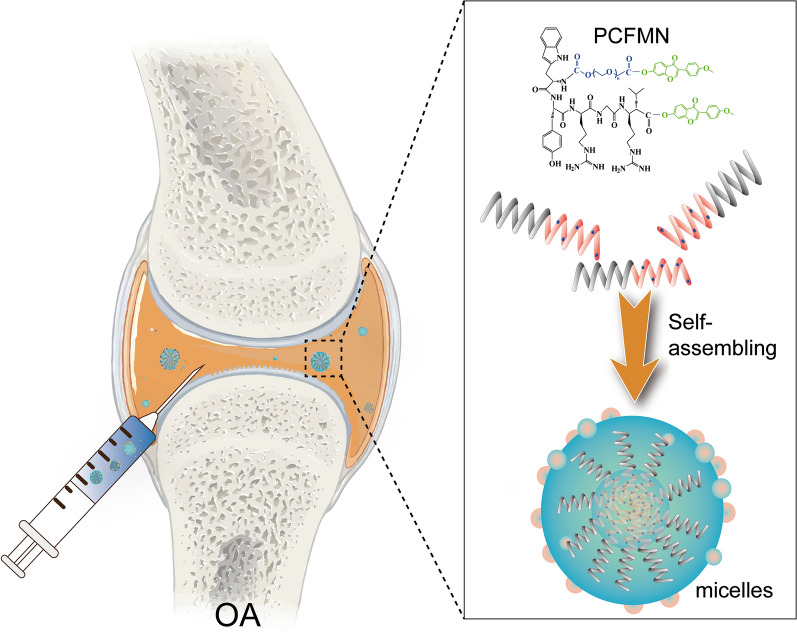

## Introduction

Characterized by synovial inflammation and cartilage destruction, osteoarthritis (OA) is a chronic and irreversible degenerative disease, which occurs commonly in elderly people [1]. Although many clinical therapeutics like nonsteroidal anti-inflammatory drugs (NSAIDs), glucocorticoids (GCs), and other drug treatments are one of the effective strategies for OA [2], there are still some shortcomings that need to be overcome such as frequent injection, gastrointestinal, cardiovascular risks, and potential overdose [3]. Thus, it is imperative to find substitutes that have minimal side effects.

Traditional Chinese medicine has a history of thousands of years, which attracts the most attention in recent years. Due to their wide range of pharmacophores, high degrees of stereochemistry [4], and modicum adverse effects [5, 6], those natural small molecules extracted from Chinese herbs are regarded as promising drug resources. Formononetin (FMN) is a phytoestrogen purified from natural herbal plants (e*.*g*.*, Astragalus membranaceus, Trifolium pretense). It was reported to have series of pharmacological effects including anti-inflammatory [7, 8], anti-angiogenic [9], antioxidant [10], cardioprotective [11], neuroprotective [12], and other effects [13], which has been extensively applied in treatment of various human diseases, such as diabetic retinopathy, Alzheimer’s disease and other diseases [14–16]. In recent years, it is found that FMN can effectively decrease proteoglycan loss and improve the pericellular matrix formation of chondrocytes [7], which may be developed as potential agents for OA treatment. However, the bioavailability of FMN is low because it has poor water solubility and can hardly penetrate through the dense matrix of cartilage [17]. Besides, with lacking specific targeting, FMN may be rapidly cleared in the joint. Sometimes, the retention time after intra-articular (IA) injection is short [18] and repeated articular injection is inevitable. Thus, it is imperative to increase the water solubility and cartilage-targeting effect of FMN to improve its pharmacological effects.

Polymer-drug conjugates (PDCs) by linking hydrophilic polymers with drugs to form uniform-sized nanoparticles is one of the effective strategies in drug synthesis to enhance drug solubility and efficacy [19]. Polyethylene glycol (PEG) that is approved by FDA with negligible toxicity and immunogenicity [20, 21] has been widely applied in PDCs, which increased drug solubility and improved cell growth [22]. Cho et al. [23] reported that PEGylation with doxorubicin contributed to effective drug delivery, prolonged nanoparticle circulation, and significantly reduced clearance rate (decreased by 73%) compared with doxorubicin without modification. After crosslinked with PEG, the release time of triptolide in plasma has been remarkably prolonged, leading to an enhanced anti-cancer effect [24]. For cartilage-targeting, peptide-mediated (e.g. chondrocyte-affinity peptide CAP, anti-inflammatory peptide KAFAK [25, 26]) and RGD-modified delivery systems [27] have been used for modification of free drug molecules. Rothenfluh et al. found the ligand WYRGRL (a Coll-II α_1_ chain-binding peptide, CollBP) can specifically bind to collagen II that is specific for cartilage matrix [28]. An in vitro study reported that WYRGRL-dexamethasone conjugate exhibited increased cartilage-targeting ability, leading to enhanced anti-inflammatory effects as compared with dexamethasone alone [29].

**Fig. 1:**
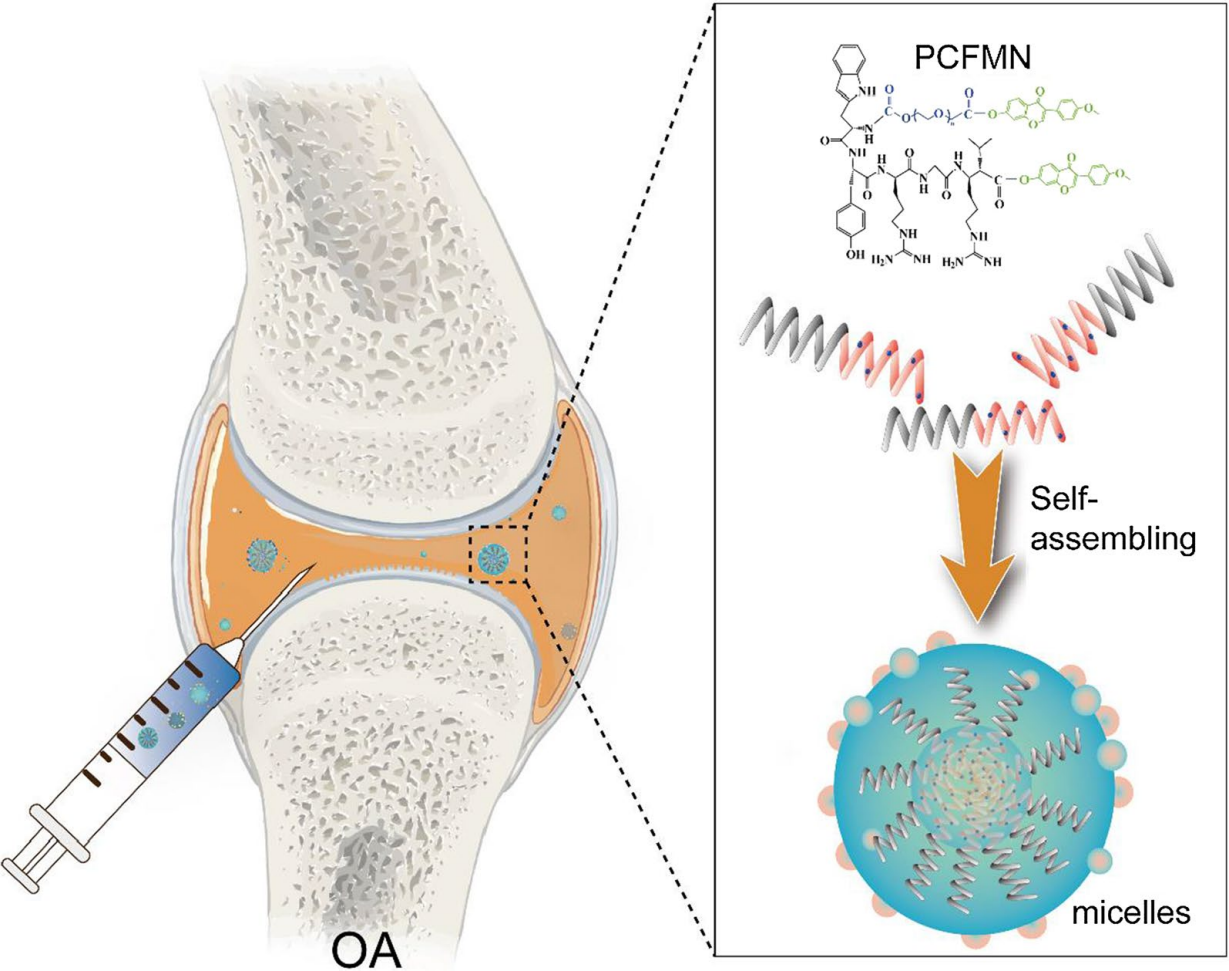
Illustration of cartilage targeted polyethylene glycol-formononetin conjugate assembling to form nano-sized drug delivery vehicles

Based on FMN that is the natural anti-inflammatory agent, we synthesized a nanosized amphiphilic polymer-drug conjugate (PEG-CollBP-FMN, PCFMN) for OA therapy, which was prepared by PEGylation of FMN followed by coupling with cartilage-targeting peptide (CollBP) to increase the bioavailability of FMN (Fig. 1). Then we investigated the anti-inflammatory effects of PCFMN on IL-1β stimulated chondrocytes and OA rat joints, as compared with unmodified FMN. This study may provide new insight into the design of a novel agent in OA therapy.

## Materials and methods

### Synthesis of PCFMN

**Fig. 2:**
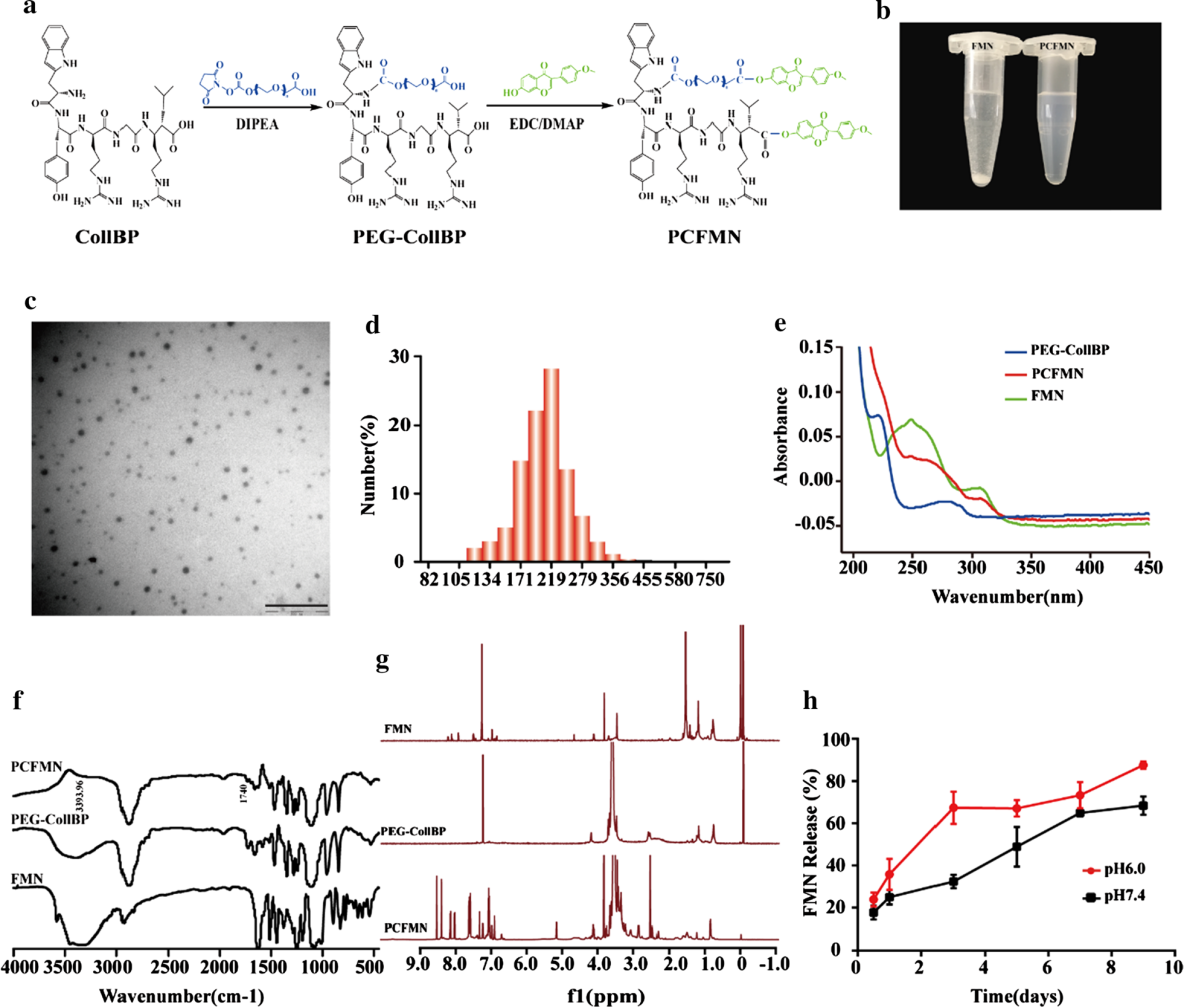
Characterization of PCFMN. **a** The Synthesis routes of PCFMN. **b** The representative digital photos of FMN (left) and PCFMN (right) aqueous solution after 3 days of storage. **c** TEM images of PCFMN. **d** Size distribution of PCFMN based on dynamic light scattering. **e** UV–Vis absorbance of different samples PCFMN, PEG-CollBP, and FMN. **f** FTIR spectra of PCFMN, PEG-CollBP, and FMN. **g** Typical ^1^H NMR spectra showing the successful crosslink formation between PEG-CollBP and FMN. **h** Cumulative drug release curves of PCFMN at different pH values (n = 3,mean $\pm$ SD)

The PCFMN was synthesized through a simple chemical process. To obtain cartilage-targeting products PEG-CollBP, 50 mg NHS-PEG-COOH (Mw, 3400 Da, Ruixi Bio, Xi’an, China) and 40 mg CollBP (WYRGRL, GL Biochem, Shanghai, China) were dissolved in 1 mL N, N-dimethylformamide (DMF) (Energy Chemical, Shanghai, China) followed by the addition of 20 μL DIPEA (Aladdin, Shanghai, China). The mixture was stirred overnight. After that, the solution was purified by dialysis (Mw = 1500 Da) with deionized water to remove excess CollBP and DMF, and lyophilized for further use. Then, the carboxyl of PEG-CollBP was activated by *N*-(3-dimethyl aminopropyl)-*N*’-ethyl carbodiimide hydrochloride (EDC)/dimethyl aminopyridine (DMAP) and reacted with the hydroxy of FMN to synthesize PCFMN by esterification reaction. Firstly, EDC and DMAP were mixed with PEG-CollBP in DMF (molar ratio of PEG-CollBP: EDC: DMAP is 1:1.1:0.1). Half an hour later, the FMN solution was slowly added to the above mixture, followed by stirring at room temperature for 48 h. Next, the products were dialyzed (Mw = 1500 Da) for 48 h and lyophilized (Fig. 2a). Finally, to obtain fluorescent nanoparticles, 2 mg fluorescent probe DID (1,1’-dioctadecyl-3,3,3’,3’-tetramethylindodicarbocyanine, 4-chlorobenzenesulfonate salt) and 5 mg PCFMN or PFMN (without cartilage-targeting peptide) were dissolved in dimethyl sulfoxide (DMSO) and stirred overnight. The whole procedure was performed in dark. And the final products were obtained after dialysis 48 h and lyophilization.

### Characterization of PCFMN

Firstly, to study the solubilization and stability of PCFMN, 5 mg FMN and 5 mg PCFMN were dissolved in 1 mL of ultrapure water, respectively, followed by ultrasound for 15 min and station for 3 days, and then photographed for observation. The morphology of PCFMN was determined by using transmission electron microscopy (TEM) (Hitachi, Japan). The size distribution of PCFMN in aqueous suspension was measured by using Malvern Zetasizer Nano ZS90, and all measurements were carried out at 25 °C. The PCFMN was confirmed by ultra-violet and visible spectrophotometer (UV–Vis, TV-1901, Beijing), FTIR spectrum (PerkinElmer, Spectrum100, USA), and ^1^H NMR (AVANCE III HD600, Zurich, Switzerland).

### The content and the release rate of FMN in PCFMN

To determine the content of FMN in PCFMN, PCFMN (200 μL, 1 mg/mL) was dialyzed in a 10 mL buffer containing 1% Tween 80 solution and esterase (20 U/mL) at 37 °C for 5 days. The content of FMN in the release medium was determined by HPLC, then the loading content of FMN can be calculated by the mass of FMN dividing the mass of PCFMN. To determine the release rate of FMN in PCFMN. The PCFMN(1 mL,1 mg/mL) was added into dialysis, then respectively immersing dialysis in 10 mL PBS (pH 7.4, pH 6.0) containing 1% Tween 80 and keeping them shaking at 37 °C. At the pre-set time, 1 mL release media was removed and replaced by 1 mL fresh buffer. We can calculate the cumulative release rate of FMN by HPLC.

### Culture of chondrocytes

Primary chondrocytes were harvested from a 5-day-old Sprague Dawley rat. Curtly, cartilage was obtained from the joints of rats’ limbs and cut into small pieces of nearly 1 mm^3^ size. The cartilage debris was digested by 0.25% trypsin at 37 °C for 30 min, and 0.2% type II collagenase (Solarbio, Beijing, China) for 4 h. Cells were collected by centrifugation (1000 rpm, 5 min) and cultured in Modified Eagle’s medium (Solarbio, Beijing, China) containing 10% (v/v) fetal bovine serum (Sijiqing, Zhejiang, China) and 1% (v/v) penicillin/streptomycin (Solarbio, Beijing, China) in an incubator with 5% CO_2_ at 37 °C. Cells were passaged when reaching nearly 80%-90% till second-generation for further research.

### Cytotoxicity studies

The cytotoxicity of PCFMN was measured by MTT assay. Briefly, the chondrocytes were seeded in a 96-well plate at a density of 6000 cells per well. After cell adherence, 200 μL of FMN or PCFMN of different concentrations was incubated for 24 h. Then, 15 μL of MTT was added to each well. After another 4 h of incubation, all the medium was removed and then DMSO (150 μL) was added to each well. Finally, the optical density at 490 nm was measured by a microplate reader (Thermo Scientific Multiskan GO Microplate Spectrophotometer). Also, the MTT assay was used to detect the effects of FMN and PCFMN on the proliferation of chondrocytes induced by IL-1β. The chondrocytes were cultured in a 24-well plate with 1.5 × 10^4^ cells per well for 24 h, then stimulated with IL-1β (10 ng/mL) to constructed an osteoarthritis model in vitro. Finally, treated with the FMN (1.25 μg/mL), PCFMN (1.25 μg/mL) or PCFMN (14 μg/mL, equivalent to 1.25 μg/mL FMN, as shown in the result section) for another 24 h. The next steps were similar to the MTT method.

### In vitro cellular uptake

As the nanoparticles carry red fluorescence dye for cell membrane, cellular uptake of PCFMN and FMN can be detected by fluorescence inversion microscope (Olympus, Japan). Chondrocytes were cultured in 24-well plates (1 × 10^4^ cells/well). When cells were fully attached, fresh medium containing PFMN-DID (without cartilage-targeting peptide) (10 μL, 1 mg/mL) and PCFMN-DID (10 μL, 1 mg/mL) fluorescent nanoparticles were added as a substitute for the old medium. After 24 h incubation, cold PBS and 4% paraformaldehyde were respectively used to wash cells and then fix for 20 min. Following, chondrocyte nuclear were stained by DAPI for 15 min. Finally, the plate was analyzed by a fluorescence inversion microscope (Olympus, Japan) to obtain corresponding fluorescence images.

### In vitro anti-inflammatory activity

To examine the effect of FMN and PCFMN on the proliferation of normal chondrocytes or IL-1β-induced chondrocytes, the chondrocytes were plated in 6-well and 24-well plates of 8 × 10^4^ cells or 1.5 × 10^4^ cells per well. After stimulation with IL-1β of 10 ng/mL for 12 h, the FMN (1.25 μg/mL), PCFMN (1.25 μg/mL), or PCFMN (14 μg/mL) were added to incubate with cells for 24 h, while cells in the normal group were untreated. Then, cells were fixed and stained by hematoxylin and eosin staining (Solarbio, Beijing, China). Immunofluorescence (IF) staining of OA-specific marker, MMP-13, was used to detect the anti-inflammatory ability of FMN and PCFMN. In the immunofluorescence (IF) test, samples were incubated with primary antibody against MMP-13 (Boster, Wuhan, China, 1:200) overnight at 4 °C. The second antibody FITC- anti-rabbit IgG (Boster, Wuhan, China) was then added for incubating with samples one more hour at room temperature in dark. Finally, the samples were treated with DAPI for nuclei stained. Images were captured using a fluorescence inversion microscope (Olympus, Japan).

### qRT-PCR detection

Chondrocytes were seeded in 6-well plates to obtain total RNA. The mRNA expression level of the cartilage-specific maker (e.g. Col2a1) and some gene expressions of catabolic markers of OA (e.g. Il-1β, Mmp-13, and Mmp-3) were analyzed by the real-time quantitative polymerase chain reaction (qRT-PCR). An RNA isolation kit (Megentec, Guangzhou, China) was used to harvest the RNA according to its manufacturer’s protocol. The reverse transcription was conducted by using a reverse transcription kit (Fermentas Company, USA). All qRT-PCR reactions were performed by a light Cycle 96 system (Roche, Switzerland) under the conditions of 10 min at 95 ℃, followed by 40 cycles of 10 s at 95 °C and then 60 s at 60 °C. The 2^−ΔΔCt^ method was used to calculate the relative gene expression levels. The primers of qRT-PCR are summarized in Table 1.

**Table 1:** Primers for qRT-PCR performance

Gene	Forward primer (5'-3')	Reverse primer (5'-3')	Size
MMP-13	GGACAAAGACTATCCCCGCC	GGCATGACTCTCACAATGCG	20
MMP-3	GTTCTGGGCTATACGAGGGC	TTCTTCACGGTTGCAGGGAG	20
IL-1β	GCACAGTTCCCCAACTGGTA	GGAGACTGCCCATTCTCGAC	20
Col2al	GACTGTGCCTCGGAAGAACT	TCTGGACGTTAGCGGTGTTG	20
GAPDH	TCCAGTATGACTCTACCCACG	TCTGGACGTTAGCGGTGTTG	20

### Induction of osteoarthritis in the rat

With the ethical approval of the Institutional Ethics Committee of Guangxi Medical University (October 25, 2018), a total of thirty Sprague Dawley rats (male, 180–200 g) were experimented in vivo. We used an anterior cruciate ligament transection (ACLT) method to establish the OA model, and all rats were randomly divided into three groups (Saline, FMN, and PCFMN groups). 4 weeks after surgery, each knee joints of the three groups was respectively injected 0.5 mL of saline, FMN (1.25 μg/mL) or PCFMN (1.25 μg/mL) once per week for 4 or 8 weeks under the same conditions. A normal group underwent a sham operation, that the rats were conducted the same surgery except for cutting the anterior cruciate ligament, meanwhile, with no further injection.

### In vivo optical imaging

To analyze the nanodrug retention time in vivo, we used an in vivo Multispectral Imaging System (Bruker, Germany) to detect the fluorescence signals of PCFMN and PFMN labeled with DID in the knee OA model. After IA injection, fluorescence imaging was conducted (n = 3). Images were obtained at corresponding time points (0, 1, 3,7, 19 d).

### Anti-inflammatory effect in vivo

After the treatment for 4 and 8 weeks, all rats were euthanized with excessive anesthesia and their knee joint samples were obtained. The repaired articular cartilages were harvested. Blinded to the treatment groups, three independent observers (SC, PYF, XF) conducted the macroscopic evaluation and a nine-area grid of each medial and lateral tibia plateau was used to evaluate the grade of articular cartilage surface (scale of 0–8) [30]. Moreover, the repaired knee joints obtained from the rats were collected and fixed with 4% formaldehyde. Then, after decalcification for one month, the joints were embedded in paraffin and sliced into the thickness of 5 μm. With sections dewaxed, histological analysis (H&E, Safranin O, and immunohistochemistry staining for MMP-13) was conducted. Then, images were captured on an optical microscope. The OARSI cartilage OA histopathology grading system was adopted to grade the repaired tissues [31].

### Statistical analysis

All the data were reported as the means ± standard deviations from at least three repeated experiments. Comparison between OA and treatment groups was examined by one-way analysis of variance (ANOVA). *P* < 0.05 was considered statistically significant.

## Results

### PCFMN synthesis and characterization

The synthetic approach for the PCFMN based on amide and esterification reaction is outlined in Fig. 2a. Comparing with FMN suspension in ultra-pure water, PCFMN exhibited favorable drug solubilization with sufficiently dissolved (Fig. 2b), which might be attributed to the presence of PEG-CollBP. Homogeneously distributed nanospheres of PCFMN were observed through TEM, and DLS analysis showed its average diameter of 218 nm (Fig. 2c, d). The successful synthesis of PCFMN was further confirmed by UV–Vis and FTIR, as shown in Fig. 2e, f. The UV–Vis-spectra of the PCFMN showed the typical absorbance peaks from both PEG-CollBP and FMN, indicating that these two components successfully interacted with each other. In FTIR spectra of PCFMN, the hydroxyl group of PEG-CollBP and FMN in 3393.96 cm^−1^ (-OH) disappeared in PCFMN and appeared in 1740 cm^−1^ (− CO −), suggesting that PCFMN was successfully synthesis through the hydroxyl esterification reaction between the carboxyl group in PEG-CollBP and FMN. The ^1^H NMR spectra of PCFMN showed the major peaks of the benzene rings in FMN at 7.0–7.5 ppm along with enhanced peaks of amide bonds at 3.5 ppm (Fig. 2g). The loading rate of FMN in PCFMN is nearly 9% by quantified with HPLC. Furthermore, the release of FMN was simulated in vitro. As showed in Fig. 2h, With the pass of time, the released FMN gradually increased. At pH 6.0, similar to the microenvironment of osteoarthritis, the cumulative release of FMN was up to 87.5% on the 9th day. However, at pH 7.4, the cumulative release of FMN was just 68.4% at the same time point.

### PCFMN showed cell-protective of OA chondrocytes in vitro

**Fig. 3:**
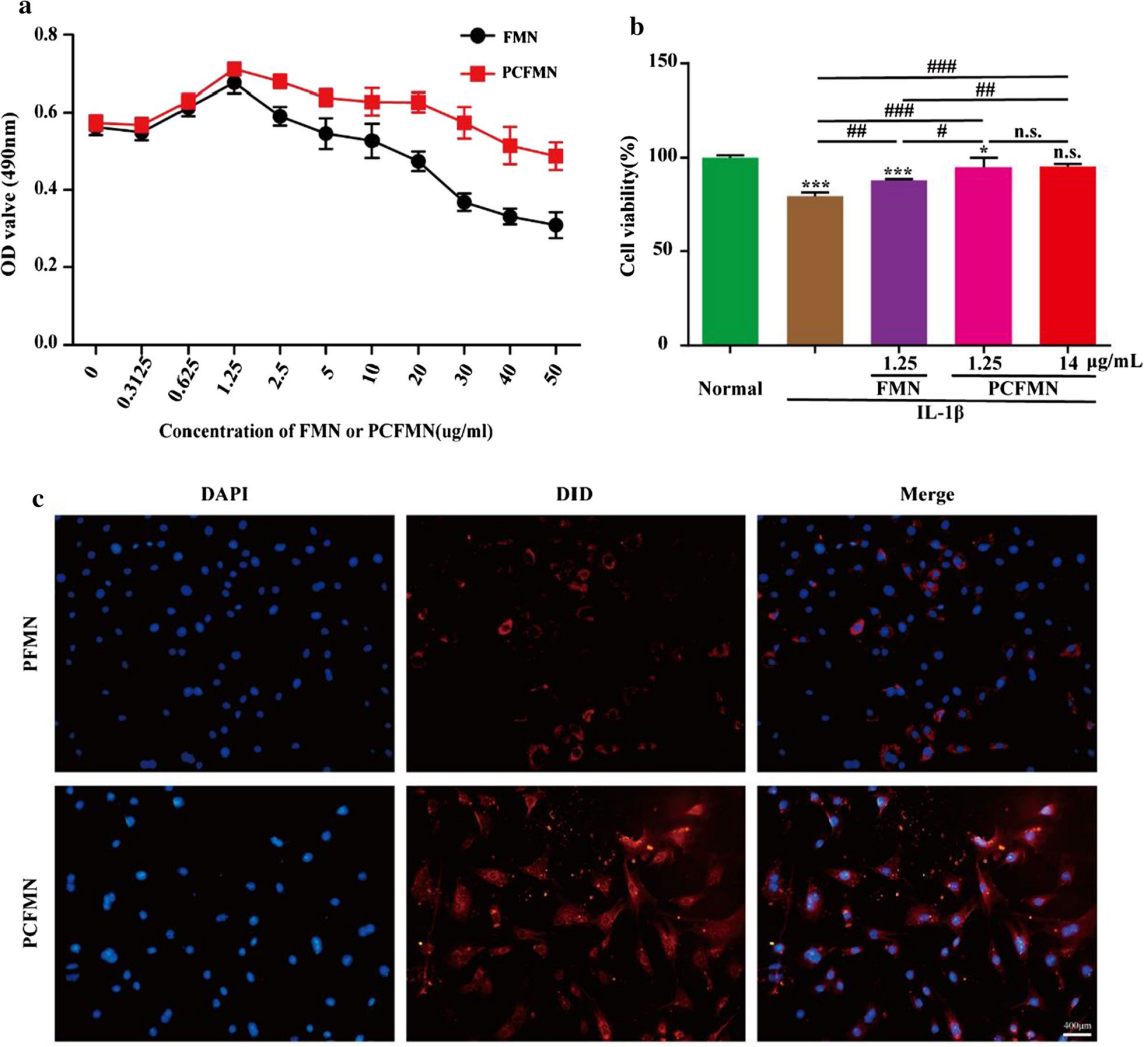
Cytotoxicity and cellular uptake of FMN or PCFMN. **a** Cell cytotoxicity of FMN and PCFMN against chondrocytes was detected by the MTT assay. **b** The effects of FMN and PCFMN on the proliferation of chondrocytes or IL-1β-induced chondrocytes after incubation for 24 h. **c** The cell uptake of PFMN or PCFMN in normal chondrocytes. Carrying DID (red), PCFMN was more effective binding to chondrocytes in vitro (blue from DAPI stain) than PFMN. Scale bars: 400 µm. (Values are the means ± SD, n = 3; *, # indicates *P* < 0.05, ##, indicates *P* < 0.01, ***, ### indicates *P* < 0.001, n.s., not significant. * is the statistical difference between the treatment group and the normal group, and # is the statistical difference between the pairwise comparison among the treatment group.)

The in vitro cytotoxicity of PCFMN against chondrocytes was investigated by MTT assay. PCFMN showed no toxicity to chondrocytes in the concentration (0–50 μg/mL), while FMN showed obviously toxicity to chondrocytes when the concentration over 20 μg/mL, and FMN of 1.25 μg/mL exhibited the highest cell proliferation (Fig. 3a) So, FMN (1.25 μg/mL), PCFMN (1.25 μg/mL), and PCFMN (14 μg/mL, equivalent to 1.25 μg/mL FMN) was chosen for further studies. The cell-protective effect of PCFMN was analyzed by the MTT method after co-cultured with these nanoparticles in IL-1β-induced OA chondrocytes for 24 h. IL-1β decreased cell viability by 20.57% compared with the normal group (Fig. 3b). Compared with the IL-1β group, through FMN increased cell viability by 8.54%, PCFMN (1.25 μg/mL) and PCFMN (14 μg/mL) treatment respectively showed a stronger chondroprotective effect with 15.46% and 16.66%r eversion, which were close to the normal control.

### Cellular uptake of PCFMN

PCFMN was confirmed by the cellular uptake analysis after treated for 24 h. Because PFMN (without cartilage-targeting peptide) and PCFMN carried DID probe, cells would be in red under the fluorescence microscope in case of the drugs were attached to or endocytosed by chondrocytes. The results of DAPI staining (blue ones) showed that the number of cells in the PFMN and PCFMN groups is similar. However, compared with the PFMN group, the PCFMN group had more obvious red fluorescence, indicating an effective cellular uptake of PCFMN (Fig. 3c).

### In vitro anti-inflammatory activity of PCFMN

**Fig. 4:**
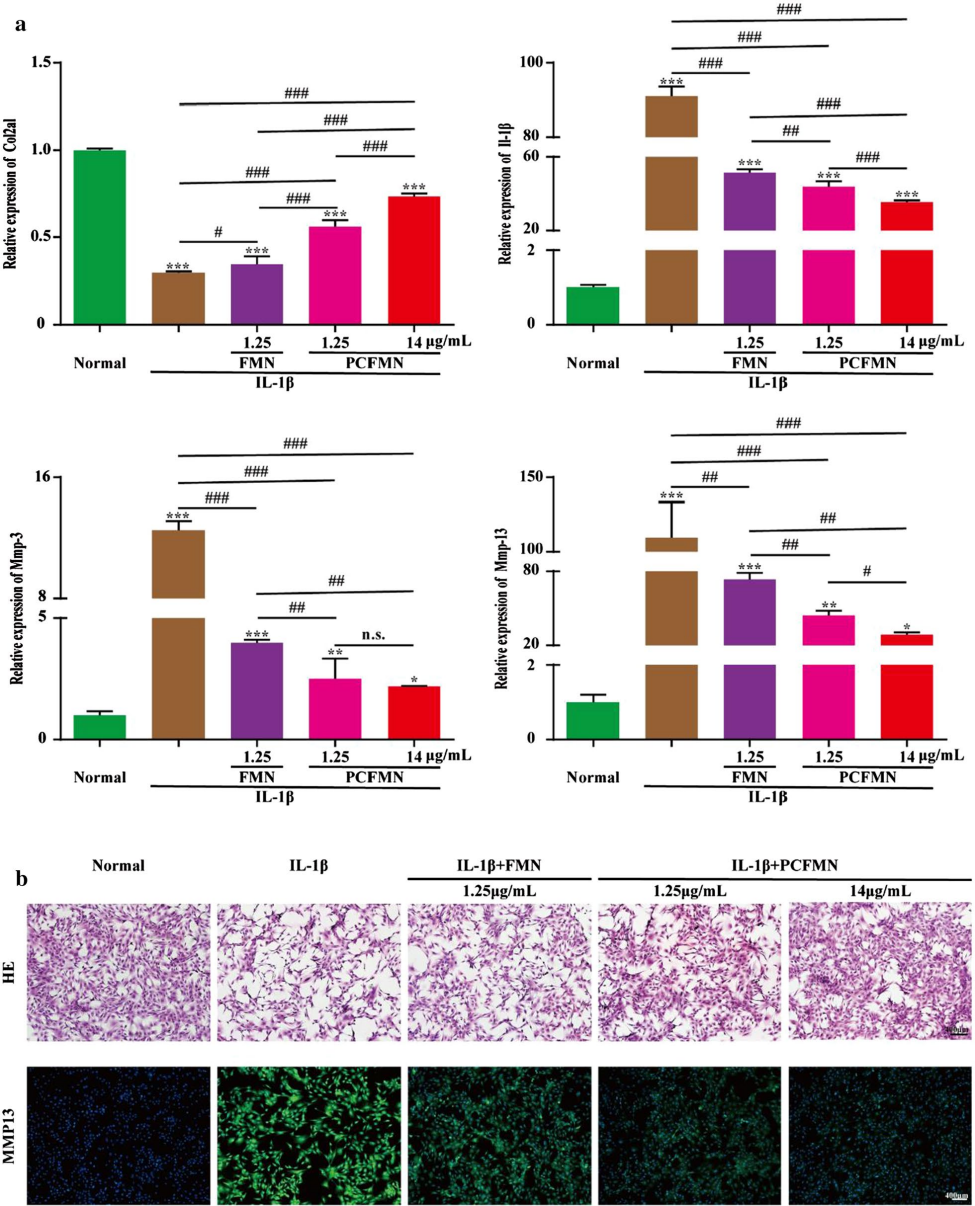
Anti-inflammatory effects of PCFMN nanoparticles in vitro*.* **a** Results of qRT-PCR showed that PCFMN caused IL-1β, MMP-3, and MMP-13 to decrease and Col2al to increase. **b** HE and immunofluorescence images suggested that PCFMN treatment resulted in MMP-13 reduction of IL-1β-stimulated chondrocytes. Scale bar: 400 μm. (Values are the means ± SD, n = 3; *, # indicates *P* < 0.05, **, ## indicates *P* < 0.01, ***, ### indicates *P* < 0.001, n.s., not significant. * is the statistical difference between the treatment group and the normal group, and # is the statistical difference between the pairwise comparison among the treatment group.)

To confirmed the anti-inflammatory activity of PCFMN on OA chondrocytes, we studied the relative expression of mRNAs, such as cartilage-specific maker (Col2a1) and OA catabolic markers (Il-1β, Mmp-3, and Mmp-13). Compared with the normal group, the expressions of Il-1β, Mmp-3 and Mmp-13 were significantly increased by 90.9, 12.5, and 109.6 times respectively in the IL-1β group (Fig. 4a). After treated with FMN (1.25 μg/mL), PCFMN (1.25 μg/mL), or PCFMN (14 μg/mL), these inflammatory factors all decreased, while PCFMN performed better in reducing the up-regulation of inflammatory factors and high concentration of PCFMN performed the best. In contrast, PCFMN (1.25 μg/mL) caused a significant increase in Col2al, similarly, the PCFMN (14 μg/mL) group upgraded Col2al the most. In addition, the morphology of chondrocytes was also observed after co-cultured with FMN (1.25 μg/mL), PCFMN (1.25 μg/mL), or PCFMN (14 μg/mL) for 24 h. Treated with IL-1β, chondrocytes were transformed into elongated and fibroblast-like ones and losing the typically fusiform-like shape (Fig. 4b). But PCFMN treatment reversed the morphology of most IL-1β treated chondrocytes to be polygonal and round shape, the effect of the high concentration of PCFMN was obvious. FMN can also recover several chondrocytes, but the recovered ratios were much lower than that induced by PCFMN. The secretions of MMP-13 performed a vital part in OA. Results of immunofluorescence staining showed that positive-staining of MMP-13 was observed in the IL-1β group. The expression of MMP-13 in three experimental groups all decreased, and the effect of PCFMN (14 μg/mL) was optimal. These results suggested that PCFMN especially in the high concentration exhibited a better anti-inflammatory against OA chondrocytes.

### The retention time of nanodrug in OA joints

**Fig. 5:**
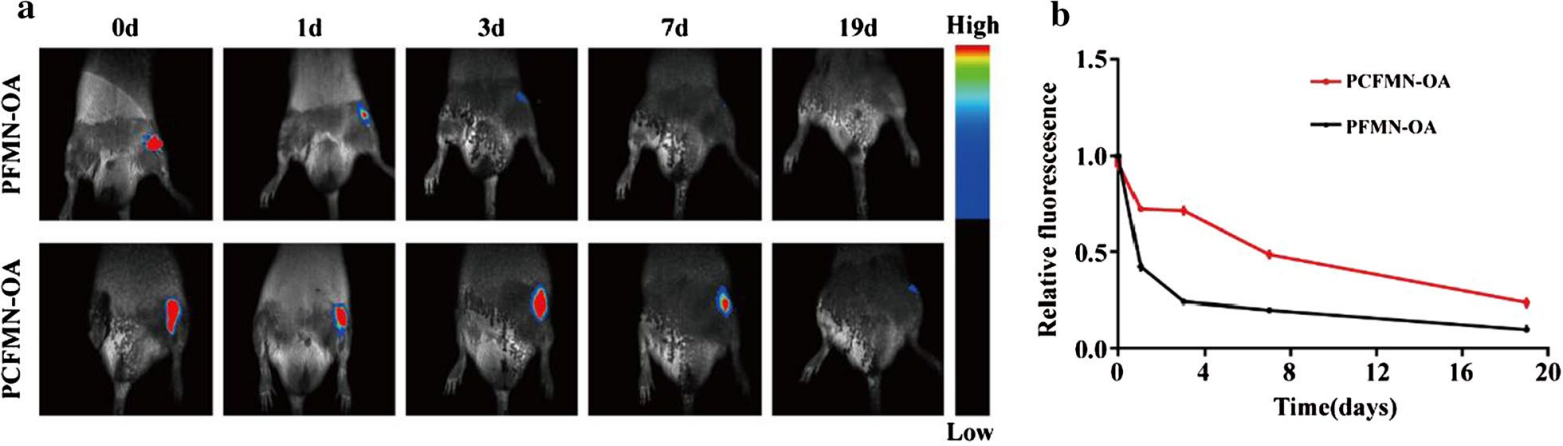
Joints retention of PCFMN and PFMN in vivo. **a** In vivo fluorescence imaging of OA rats after IA-injection of DID-labeled PCFMN and PFMN for 0, 1, 3, 7 and 19 days. **b** Quantitative analysis for fluorescence of PCFMN or PFMN in the joints after IA-injection for 0, 1, 3, 7 and 19 days (relative fold-changes to the first-time point were calculated and shown as mean ± SD, n = 3)

An in-vivo imaging system was used to detect the retention times of PCFMN and PFMN in the OA joints on days 0,1,3,7 and 19. As shown in (Fig. 5a, b), at different time points, the fluorescence intensity in the PCFMN group was higher than that in the PFMN group. However, PFMN showed drops in the fluorescence intensity with time past and no obvious fluorescence was observed on day 19.

### PCFMN attenuated the progression of OA in vivo

After treated with PCFMN, rats were sacrificed at 4 and 8 weeks respectively. We conducted macroscopic scoring, hematoxylin–eosin (H&E) staining, Safranin O-fast green staining, and immunohistochemistry staining of MMP-13.

**Fig. 6:**
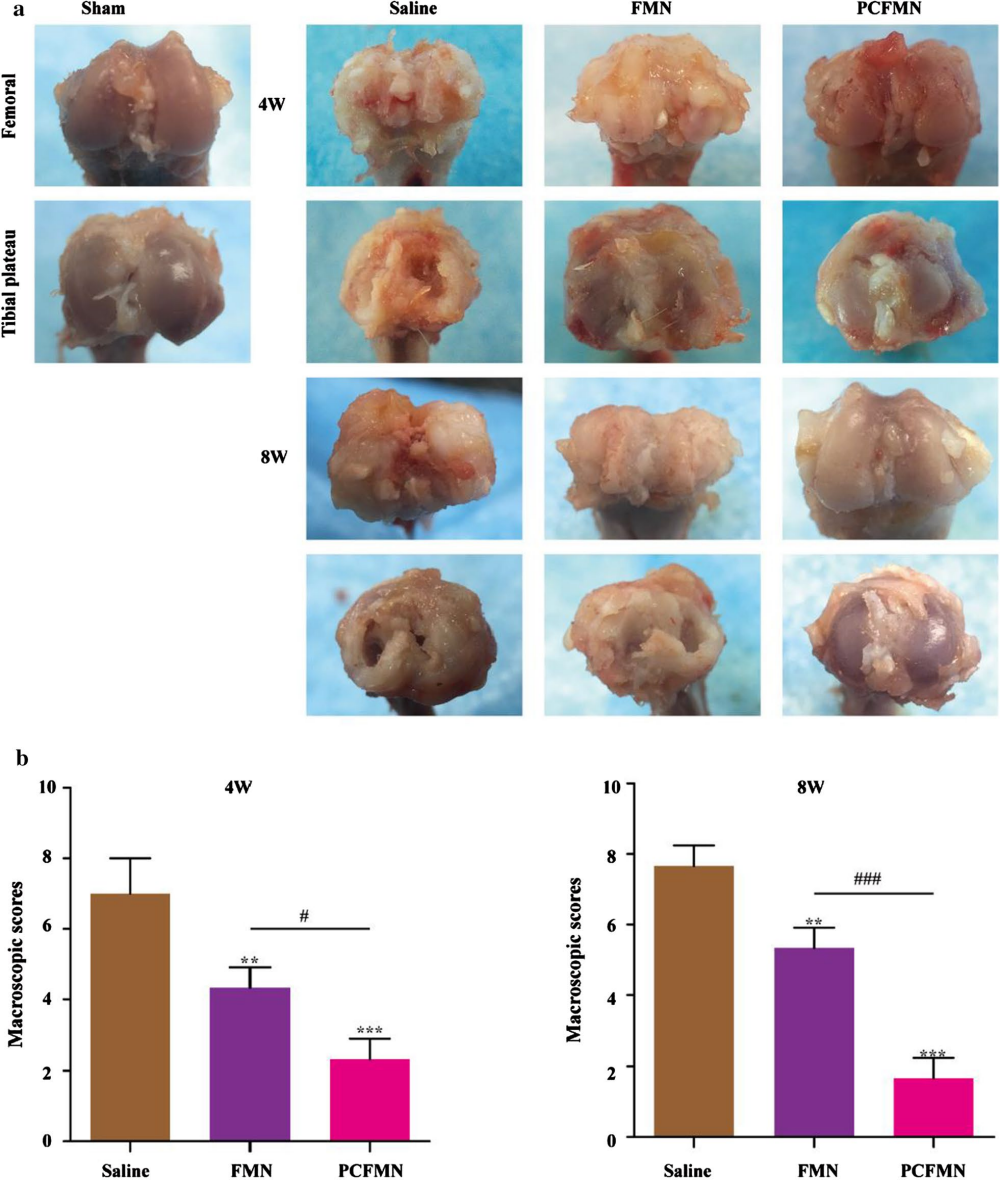
The macroscopic observation (**a**) and macroscopic score (**b**) of cartilage after IA-injection with Saline, FMN, and PCFMN for 4 and 8 weeks. (Values are the means ± SD, n = 5; # indicates *P* < 0.05, ** indicates *P* < 0.01, ***, ### indicates *P* < 0.001. * is the statistical difference between the FMN, PCFMN group and the saline group, and # is the statistical difference between the FMN and PCFMN group.)

Compared with the sham group, OA characteristics, including cartilage erosion, osteophyte formation, and deterioration over time were observed in the saline-treated group (Fig. 6a). After 4 and 8 weeks of administrating with PCFMN, reducing osteophytes and lesion surface was observed and scores declined to 66.67% and 78.26% respectively. Although FMN also ameliorated the cartilage destruction caused by ACLT, they performed poorer than PCFMN.

**Fig. 7:**
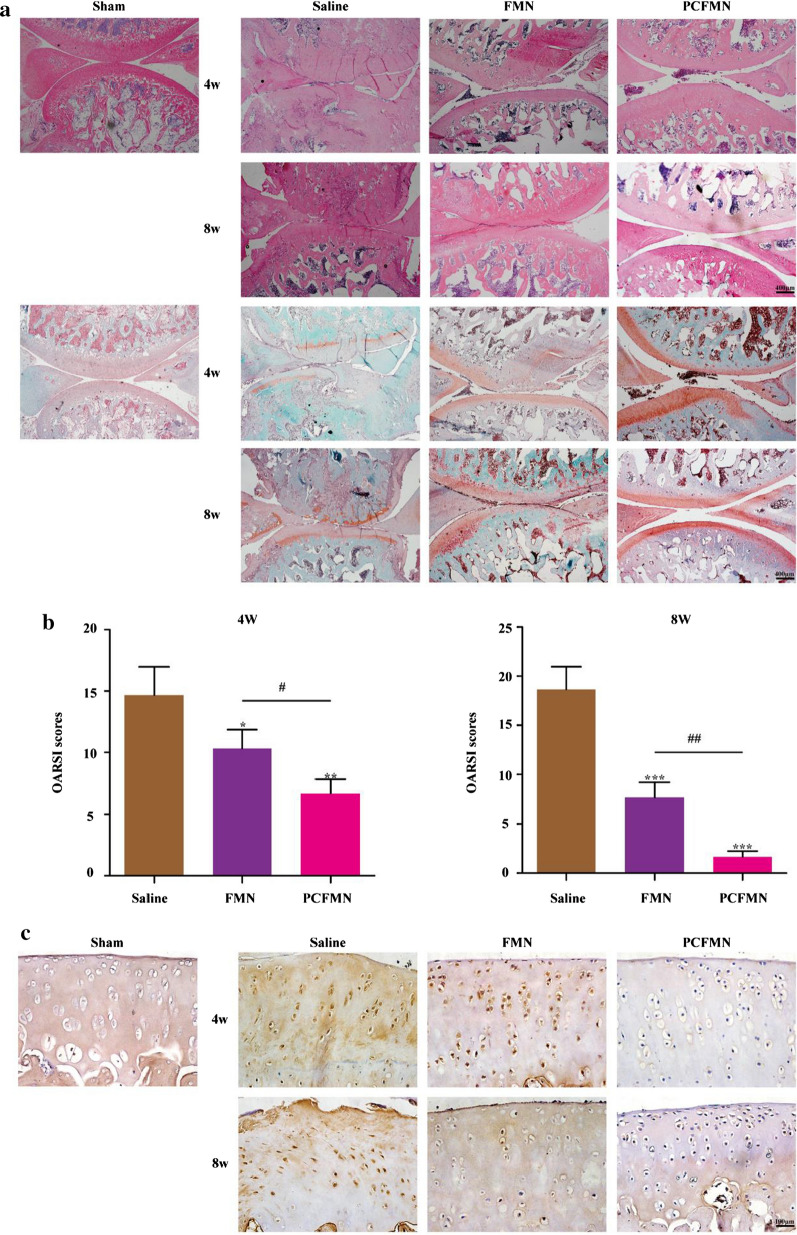
Effect of PCFMN on ACLT-induced OA. **a** Representative hematoxylin–Eosin (HE) and Safranin-O/fast green (SOFG) photomicrographs. Scale bar: 400 μm. **b** OARSI score of articular cartilage was determined. **c** Histological sections immunostained for MMP-13 on cartilage sections. Scale bar: 100 μm. (Values are the means ± SD, n = 5; *, # indicates *P* < 0.05, **, ## indicates *P* < 0.01, *** indicates *P* < 0.001. * is the statistical difference between the FMN, PCFMN group and the saline group, and # is the statistical difference between the FMN and PCFMN group.)

Following, we used hematoxylin–eosin (H&E) and Safranin O/fast green (SOFG) staining to assess the cartilage tissues of the different groups. Compared with the saline group, the other two groups presented various degrees of improvement such as proteoglycan retention, no bone erosion, and tidemark integrity promotion. Specifically, less severe lesions, decreased surface denudation, as well as increased tissue cellularity and cloning were observed in the PCFMN group, which indicated that the PCFMN group had more importance in chondroprotective effects (Fig. 7a). Furthermore, the Safranin O/fast green positive staining in the PCFMN group was significantly stronger than the other groups (Fig. 7a). All these results indicated that the PCFMN group resulted in better glycosaminoglycan deposition, cartilage matrix depletion attenuation, and overall cartilage thickness retention. OARSI score as illustrated in Fig. 7b, the PCFMN group, one of the treatment groups, grasped lower OARSI scores with about 54.54% and 90.07% reduction at week 4 and 8, respectively, compared with the PBS group. Also, through immunohistochemistry staining, the expression of MMP-13 was investigated. Positive staining (dark brown) on the cartilage surface representing MMP-13 was observed in the OA group (Fig. 7c). In contrast, the MMP-13 expression of the PCFMN group showed significant decreases than that of the FMN group and approximately identical to sham control.

## Discussion

Targeting nanodrug has emerged as promising therapies for OA treatment, but penetration through the dense cartilage matrix and drug retention in the joint cavity still challenged the therapeutic effects [32]. In this study, we fabricated cartilage-targeting nanodrug of PCFMN by modification of FMN by both PEGylation and coupling with cartilage-targeting peptide. Based on the in *vitro* and in *vivo* tests, it was found that PCFMN could specifically target cartilage, which has been proved by our previous research [33] and exert strong anti-arthritic and chondroprotective effects.

PCFMN showed high drug solubilization than unmodified FMN after PEGylation (Fig. 2b), demonstrating the effectiveness of PEGylation. PEGylation can enhance the pharmacokinetic properties of drugs [34]. With excellent hydrophilic ability, PEG chains grafted on nanoparticles generate a sufficiently thick hydrated cloud [35] that strongly prevents NPs from aggregation. Functionalized with low molecular weight PEG, nanomaterials have enhanced penetration ability through mucus [36] and other tissues, which facilitated drug delivery to the lung [37], breast [38], and colorectal carcinomas [39]. CollB-peptide has been demonstrated to bind to collagen II α_1_ and shows an outstanding application in drug delivery and imaging [28, 40]. As evidenced by the cellular uptake analysis (Fig. 3c), the CollB-peptide conjunction endowed PCFMN with a high cell penetration efficiency. Thus, PCFMN had a good solubility (Fig. 2g) and cellular uptake capacity (Fig. 3c), contributed to enhanced anti-arthritic effects both in vitro and in vivo.

PEGylation and targeted peptide modification make PCFMN with good anti-inflammatory and chondroprotective effects both in vitro and in vivo (Figs. 4, 5, 6, 7). As a plant natural product, FMN has antioxidant properties and shows neuroprotective effects against traumatic brain injury through activation of Nrf2-dependent antioxidant pathways [41]. Further, FMN showed anti-inflammatory capacity by suppressing IL-6 and TNF-α in neuroinflammatory rats [16] and effectively antagonized proteoglycan loss through decreasing the expression of MMP-3, MMP-13, and attenuating oxidative stress [7]. Further, with the studying of the repression of OA-marked factors, such as IL-1β, MMP-3, and MMP-13, and the promotion of cartilage-specific marker (Col2al), anti-inflammatory and chondroprotective characters of PCFMN were exhibited (Fig. 4a). Both MMP-13 and MMP-3 can result in the degradation of a variety of cartilage ECM components in OA processes [42] and MMP-13 exhibited a preference for collagen II [43]. As expected, compared with free FMN, PCFMN, a cartilage-targeting, and PEGylated nanodrug, had a more remarkable effect on down-regulation MMP-13 and preventing cartilage degradation (Fig. 7c), indicating that a targeted nanodrug design is a promising therapeutic strategy for OA.

In conclusion, the PCFMN nanoparticles with improved hydrophobic drug solubilization and cellular uptake ability increased drug accumulation and finally achieved effective anti-arthritic effects. Our study indicates that modification of drug by PEGylation and coupling with targeting peptide might be a promising strategy for OA therapy.

## Data Availability

The datasets used and/or analyzed during the current study are available from the corresponding author on reasonable request.
